# Mortality patterns in long-term survivors of childhood or adolescent central nervous system tumour in Sweden

**DOI:** 10.1007/s11060-019-03321-w

**Published:** 2019-11-01

**Authors:** Wuqing Huang, Jan Sundquist, Kristina Sundquist, Jianguang Ji

**Affiliations:** 1 Center for Primary Health Care Research, Lund University/Region Skåne, Skåne University Hospital, Jan Waldenströms gata 35, 20 502 Malmö, Sweden; 2 Department of Family Medicine and Community Health, Department of Population Health Science and Policy, Icahn School of Medicine at Mount Sinai, New York, USA; 3 Center for Community-Based Healthcare Research and Education (CoHRE), Department of Functional Pathology, School of Medicine, Shimane University, Shimane, Japan

**Keywords:** Central nervous system tumour, Long-term survivors, Mortality patterns

## Abstract

**Purpose:**

A growing number of young patients with central nervous system (CNS) tumour survived for more than five years. However, these long-term survivors might be at risk of multiple late effects thus leading to a higher risk of late mortality. We aimed to explore the risk of late mortality and the pattern of mortality among long-term survivors of childhood or adolescent CNS tumour.

**Methods:**

We identified 5-year survivors with childhood or adolescent CNS tumour before age 20 years through the Swedish Cancer Registry. Five controls were randomly matched for each patient to generate the reference group. We retrieved information about death via Cause of Death Register. We calculated the absolute excess risk (AER) of death and the hazard ratio (HR) of death using Cox proportional hazard model.

**Results:**

Long-term survivors with CNS tumour suffered a significant higher risk of overall mortality (HR 6.56, 95% CI 5.71–7.53; AER 5.89, 95% CI 5.03–6.87). The mortality rate declined with the increasing survival time, but it was still higher even after 30 years of follow-up. Malignant neoplasms contributed mostly to late mortality with an AER of 3.75 (95% CI 2.95–4.75). Female survivors, survivors diagnosed at a younger age and survivors with medulloblastoma were particularly strongly associated with a higher risk of death.

**Conclusions:**

Long-term survivors of childhood and adolescent CNS tumours are at a higher risk of late mortality, and the risk of death is affected by gender, age at diagnosis and types of CNS tumour.

**Electronic supplementary material:**

The online version of this article (doi:10.1007/s11060-019-03321-w) contains supplementary material, which is available to authorized users.

## Introduction

Central nervous system (CNS) tumour was the first most commonly newly-diagnosed type of tumour in Swedish population before age 20 years in 2016, with age-standardised incidence 4.7 per 100,000 person-years in girls and 3.7 in boys [1]. Due to the improvement of treatments and earlier detection, the five-year survival rate for patients with CNS tumour has dramatically increased; more than 70% of patients diagnosed with childhood and adolescent CNS tumour can survive beyond five years [2]. In clinical practice, the 5-year survival rate is commonly used as a benchmark of clinical cure, but the mortality rate, as well as the mortality patterns, among long-term survivors of childhood and adolescent CNS tumour are still largely unknown. It is thus highly necessary to explore this unanswered question by using high-quality data with high power to provide better clinical guidelines and surveillance recommendations of these survivors. Some previous studies have explored the overall and cause-specific mortality among long-term survivors with childhood cancers [3–10], whereas limited evidence existed on specifically investigating the patterns of death cause among survivors with CNS tumour diagnosed in children or adolescents.

In this population-based cohort study utilising a range of Swedish nationwide registers, we aimed to explore the mortality rate and the mortality patterns among long-term survivors who were diagnosed with CNS tumour before 20 years of age. In addition, we investigated whether the mortality patterns were associated with survival time and with specific types of CNS tumour.

## Materials and methods

### Study population

We identified all patients who were diagnosed with childhood and adolescent CNS tumour (age < 20 years) between 1958 and 2016 from the Swedish Cancer Registry by using the 7th International Classification of Disease (ICD-7) code 193. Only primary CNS tumour was considered in the current study. The Swedish Cancer Registry was created in 1958 and is maintained by the National Board of Health and Welfare. Clinicians, pathologists, and cytologists in Sweden must report all newly diagnosed cases of cancer to the Swedish Cancer Registry separately to complement each other, which guarantees its high sensitivity and specificity [11]. Long-term survivors are defined as those patients who have survived at least five years after the diagnosis, which means that we excluded patients who died within five years post-diagnosis.

Five cancer-free individuals were matched to each patient conditional on the same birth year, gender, birth country (Sweden or abroad) and highest education (1–9 years, 10–11 years or > 11 years). By retrieving from the Total Population Register which covers the whole Swedish population who have a residency permit and combining with the Swedish Cancer Register, individuals who did not have a diagnosis of CNS tumour during the study period and were still alive on the date of the corresponding survivors being started to follow-up were randomly selected.

The Ethics Committee at Lund University approved (February 6, 2013) this nationwide cohort study (Dnr 2012/795). The project database is located at the Center for Primary Health Care Research in Malmö, Sweden.

### Ascertain of outcomes

By linking to the Cause of Death Register, we collected death-related information, including date of death and cause of death. Causes of death were coded according to International Classification of Disease (ICD), 7th version for deaths before 1969, 8th version for deaths between 1969 and 1986, 9th version for deaths between 1989 and 1996 and 10th version for deaths after 1996. The ICD-7, ICD-8 and ICD-9 codes were translated to ICD-10 codes to keep consistent of the underlying cause of death during the study period. The main outcome was the overall mortality. To explore the patterns of death causes, we further classified the cause of deaths into nine specific subgroups: infectious and parasitic disease (ICD-10 codes: A00-B99); malignant neoplasms (ICD-10 codes: C00-D48); endocrine, nutritional and metabolic diseases (ICD-10 codes: E00-E90); diseases of the nervous system and sense organ (ICD-10 codes: F00-H95); diseases of the circulatory system (ICD-10 codes: I00-I99); diseases of the respiratory system (ICD-10 codes: J00-J99); diseases of the digestive system (ICD-10 codes: K00-K93); injury, poisoning and certain other consequences of external causes (ICD-10 codes: S00-T98); and others. To examine the temporal trends of causes of death with survival time, we divided the causes of death into two categories, neoplasm-specific death and non-neoplastic death.

### Assessment of exposure and covariates

By retrieving from the Swedish Cancer Registry, we got information about the diagnosis of CNS tumour, including date of diagnosis and histology of tumours. Based on age at diagnosis of CNS tumour, survivors were classified into four categories: survivors diagnosed at preschool age (0–4 years old), primary school age (5–9 years old), preadolescent (10–14 years old) and adolescent (15–19 years old). Survivors diagnosed before 15 years old were also noted as survivors diagnosed at childhood. According to the 2016 World Health Organization Classification of Tumours of the Central Nervous System, we classified CNS tumour into nine categories based on histology, including medulloblastoma, ependymoma, meningioma, ependymoblastoma, astrocytoma, craniopharyngioma, haemangioma, neurinoma, and others. Besides, CNS tumour was further classified based on the anatomic location: brain, spinal cord, peripheral nerve and others.

We further linked subjects to the Statistics Sweden’s Total Population Register and Population Housing Census to obtain baseline demographic characteristics which were shown in Table 1. Birth year was shown as born before or after 1980. Birth country was classified into born in Sweden or abroad. Highest education level was modeled as 1–9 years, 10–11 years and > 11 years.

**Table 1:** Characteristics of long-term survivors with childhood or adolescent central nervous system tumour and matched controls

Charateristics	Survivors of CNS tumour		Matched controls	
	No	%	No	%
Overall	3264	100.0	16,320	100.0
Total follow up time, person years	66,600		367,108	
Gender				
Female	1508	46.2	7540	46.2
Male	1756	53.8	8780	53.8
Birth year				
≤ 1980	1593	48.8	7965	48.8
> 1980	1671	51.2	8355	51.2
Birth country				
Sweden	3133	96.0	15,665	96.0
Others	131	4.0	655	4.0
Highest education				
1–9 years	563	17.3	2815	17.3
10–11 years	1936	59.3	9680	59.3
12 + years	765	23.4	3825	23.4
Age at diagnosis				
Preschool age (0–4 years old)	960	29.4		
Primary school age (5–9 years old)	746	22.9		
Preadolescent (10–14 years old)	821	25.1		
Adolescent (15–19 years old)	737	22.6		
Year of diagnosis				
1958–1990	1618	49.6		
1991–2015	1646	50.4		
Histology				
Astrocytoma	1468	45.0		
Craniopharyngiomas	175	5.3		
Ependymoma	176	5.4		
Ependymoblastoma	38	1.1		
Haemangioma	57	1.7		
Medulloblastoma	245	7.5		
Meningioma	81	2.5		
Neurinoma	149	4.6		
Others	875	26.9		
Location				
Brain	2704	82.8		
Spinal cord	206	6.3		
Peripheral nerve	277	8.5		
Others	77	2.4		
Follow-up time, years				
≤ 10	936	28.7		
11–20	842	25.8		
21–30	660	20.2		
> 30	826	25.3		

*CNS* central nervous system

The unique individual national identification number was assigned to all the residents living in Sweden for longer than three months, which was replaced by serial numbers to provide anonymity and used to link several registers in this study.

### Statistical analyses

Cox proportional hazard model was used to calculate hazard ratios (HRs) and 95% confidence intervals (CIs) between CNS tumour diagnosis and mortality. Follow-up of CNS tumour survivors were started at the date of five years after the primary diagnosis of CNS tumour, and the matched controls was started at the same date with the corresponding survivors. We censored individuals on the date of death or at the end of the study period (March 2017), whichever came first. The mortality rate was calculated as the number of deaths divided by 1000 person-years of follow-up. We further calculated absolute excess risk (AER) and its 95% CI, which was defined as the minus of mortality rate in the study population to the mortality rate in the matched controls.

We stratified analyses by gender and age at diagnosis to explore the difference of mortality patterns. In addition, we examined the trends of overall mortality, neoplasm-caused mortality and non-neoplastic causes mortality among long-term survivors who were followed up ≤ 10 years, 11–20 years, 21–30 years and > 30 years, respectively (equal to surviving for 5–15 years, 16–25 years, 26–35 years and > 35 years, respectively). We also calculated HRs and 95% CIs by comparing the mortality of specific types of CNS tumour to their matched controls.

All analyses were performed using SAS version 9.3 (SAS Institute, Cary, NC).

## Results

A total of 3264 long-term survivors of childhood or adolescent CNS tumour were identified from the Swedish Cancer Registry between 1958 and 2016. After a total of 66,600 person-years of follow-up, 471 patients had died, generating a mortality rate of 7.1 per 1000 person-years. Among 16,320 matched controls, 434 subjects had died after 367,108 person-years of follow-up with a mortality rate of 1.18 per 1000 person-years. Characteristics of long-term survivors of CNS tumour are shown in Table 1. Male survivors (53.8%) surpassed female survivors (46.2%) and 77.4% of long-term survivors were diagnosed with CNS tumour during childhood (< 15 years old). About half of them (48.8%) were born before 1980 and the majority of patients (96.0%) were born in Sweden.

We present the HR and AER among long-term survivors of childhood and adolescent CNS tumour in Table 2. As compared to matched controls, long-term survivors suffered a significantly higher risk of death with a HR of 6.56 (95% CI 5.71–7.53), which led to an excess mortality rate of 5.89 per 1000 person-years (95% CI 5.03–6.87). The mortality rate was significantly higher for all the cause-specific deaths with an exception of death due to injury, poisoning and certain other consequence of external causes. Malignant neoplasms, infectious and parasitic disease, and diseases of the nervous system and sense organ ranked the top three causes related to the increased risk of mortality, generating HRs of 15.89 (95% CI 12.42–20.32), 9.53 (95% CI 2.38–38.15) and 5.80 (95% CI 2.92–11.52), respectively. The top three causes accounted for absolute excess risk were malignant neoplasms (AER 3.75; 95% CI 2.95–4.75), diseases of the respiratory system (AER 0.49; 95% CI 0.28–0.80) and diseases of the nervous system and sense organ (AER 0.23; 95% CI 0.09–0.49).

**Table 2:** Hazard ratio and absolute excess risk with 95% CI for all-cause and specific disease mortality among survivors with childhood or adolescent central nervous system tumour and matched controls

Outcomes	Number of deaths		Mortality rates (per 1000 person-years)		HR	95% CI	AER	95% CI
	Survivors	Matched controls	Survivors	Matched controls				
All cause mortality	471	434	7.07	1.18	6.56	5.71–7.53	5.89	5.03–6.87
Specific disease mortality								
Malignant neoplasms	270	112	4.05	0.31	15.89	12.42–20.32	3.75	2.95–4.75
Infectious and parasitic disease	6	3	0.09	0.01	9.53	2.38–38.15	0.08	0.01–0.35
Diseases of the nervous system and sense organ	18	16	0.27	0.04	5.80	2.92–11.52	0.23	0.09–0.49
Diseases of the respiratory system	14	16	0.21	0.04	4.81	2.29–10.09	0.17	0.06–0.39
Endocrine, nutritional and metabolic diseases	4	11	0.06	0.03	3.90	1.05–14.54	0.03	0.00–0.16
Diseases of the digestive system	8	18	0.12	0.05	3.85	1.52–9.77	0.07	0.00–0.23
Diseases of the circulatory system	44	64	0.66	0.17	3.74	2.51–5.57	0.49	0.28–0.80
Injury, poisoning and certain other consequences of external causes	36	141	0.54	0.38	1.40	0.96–2.03	0.16	0.00–0.40
Others	37	30	0.56	0.08	7.57	4.53–12.66	0.47	0.26–0.82

*AER* absolute excess risk, *CNS* central nervous system, *CI* confidence interval, *HR* hazard ratio

**Fig. 1:**
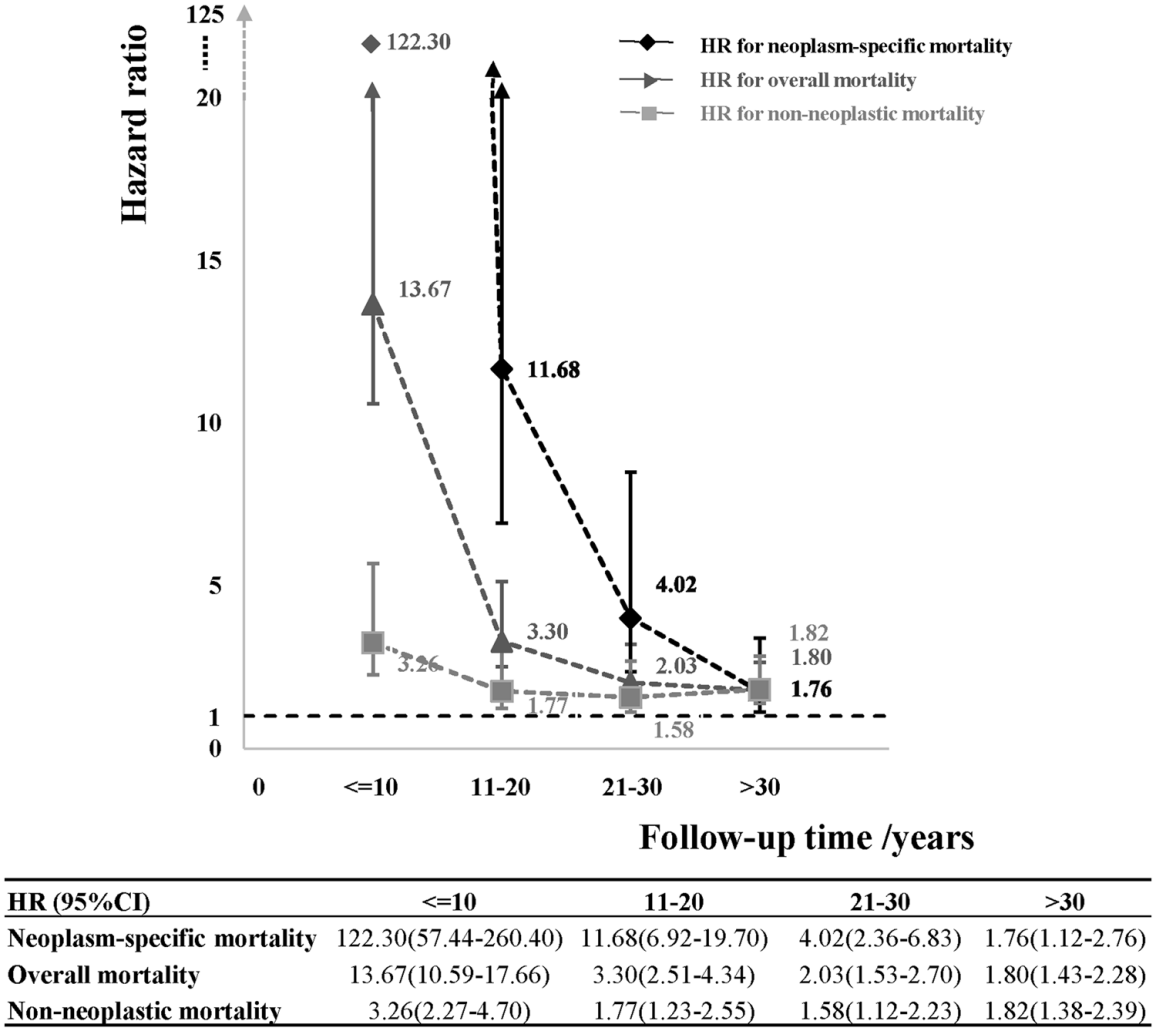
Temporal trends of hazard ratio of all-cause, neoplasm-specific or non-neoplastic mortality among long-term survivors with childhood or adolescent central nervous system tumour with the increasing survival time

We present the survival curves according to all-cause mortality, neoplasm-caused mortality and non-neoplastic mortality in Supplementary Fig. 1 (Online only). The difference between survivors and controls was more attributable to neoplasm-caused death in the earlier period of follow-up while the difference was dominated by non-neoplastic death in the latter period. We further explored the trend for all-cause mortality, neoplasm-caused mortality and non-neoplastic mortality with the increasing survival time, shown in Fig. 1. The HR significantly declined with the increasing survival time, especially for neoplasm-specific death. During the first 10 years of long-term survivors, the risk of death due to neoplasm was extremely high (HR 122.30), and it dramatically declined for the second 10 years with a HR of 3.30, and a HR of 2.03 for the third 10 years, and a HR of 1.76 for those who were followed more than 30 years. The HR for overall mortality was 13.67 at the first 10 years of follow-up, and then decreased over survival time. But the overall mortality rate was still significantly higher even after 30 years of follow-up (HR 1.80). HR for deaths caused by non-neoplastic diseases was 3.20 for the first 10 years of follow-up and kept declining until followed up for 30 years.

**Fig. 2:**
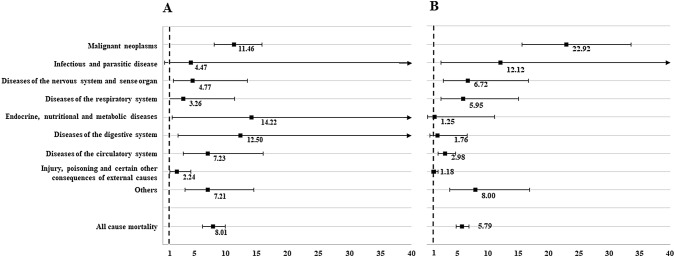
Hazard ratio and 95% confidence interval of all-cause, disease-specific mortality among long-term survivors with childhood or adolescent central nervous system tumour stratified by gender. **a** Female survivors; **b** male survivors

**Fig. 3:**
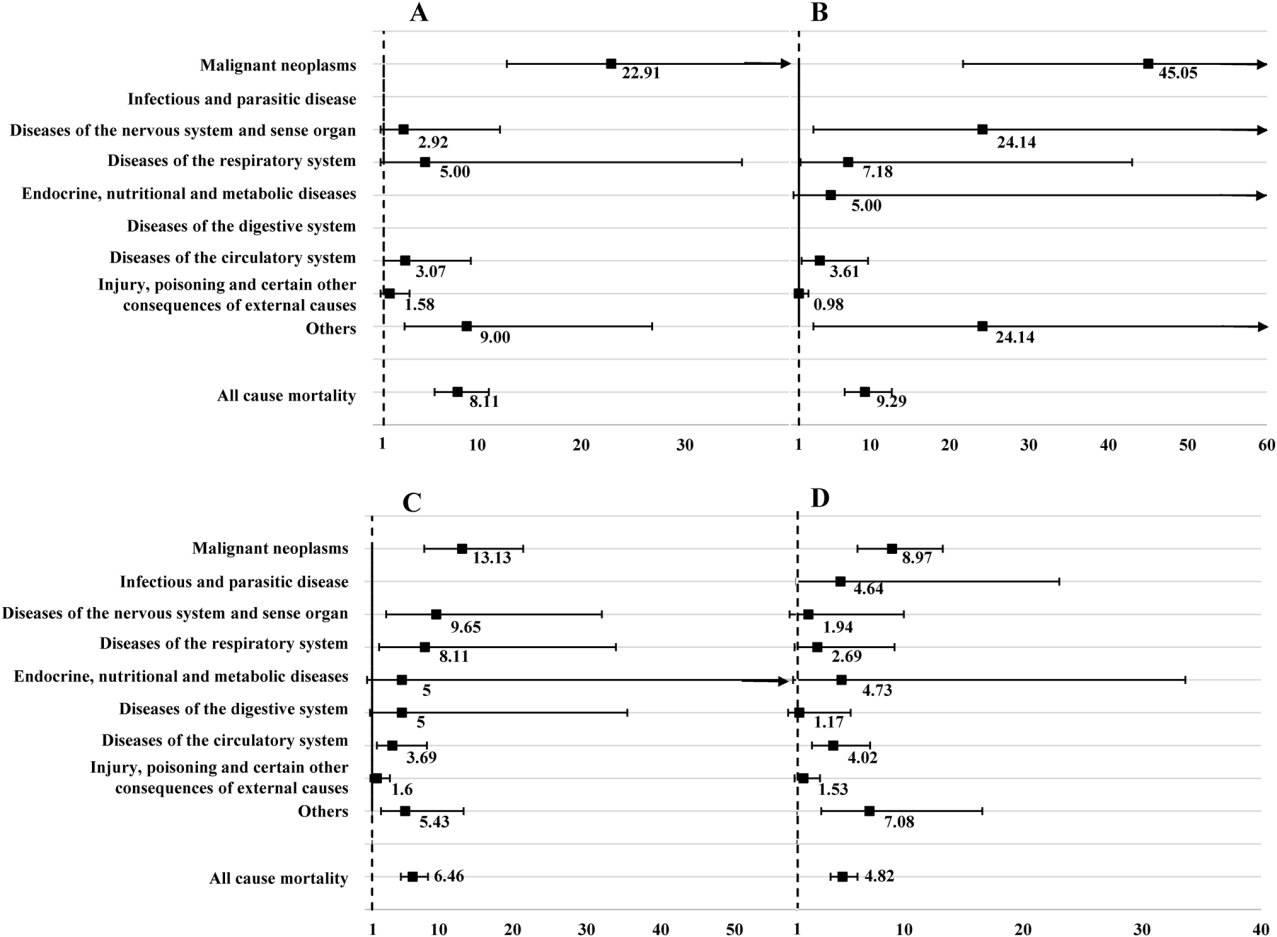
Hazard ratio and 95% confidence interval of all-cause, disease-specific mortality among long-term survivors with childhood or adolescent central nervous system tumour stratified by age at diagnosis. **a** Preschool age; **b** primary school age; **c** preadolescent; **d** adolescent

As shown in Fig. 2, analyses stratified by gender found that female survivors experienced an even higher overall mortality with a HR of 8.01, while the HR was 5.79 for male survivors. For specific causes of death, endocrine, nutritional and metabolic diseases played a predominant role among female survivors (HR 14.22), followed by diseases of digestive system (HR 12.50) and malignant neoplasms (HR 11.46). However, the highest risk of mortality in male survivors was caused by malignant neoplasms (HR 22.92), followed by infectious and parasitic disease (HR 12.12) and other causes (HR 8.00). In Fig. 3, we stratified the analyses based on age at diagnosis. The overall risk of death for survivors aged 0 to 4, 5 to 9, 10 to 14, and 15 to 19 years was 8.11, 9.29, 6.46, and 4.82 respectively. In terms of mortality patterns, both childhood and adolescent survivors were most likely to die due to malignant neoplasms (HR 22.04 vs. 8.97).

In Table 3, we present the results for specific types of CNS tumour as compared to matched controls. Survivors diagnosed with medulloblastoma were at the highest risk of late mortality with a HR of 17.64 (95% CI 10.30–30.20) and an excess mortality rate of 14.29 (95% CI 8.69–23.09) per 1000 person-years. The relative risk of death was followed by ependymoma (HR 9.52) and meningioma (HR 7.93). The excess mortality rate was followed by ependymoma (AER 10.26) and ependymoblastoma (AER 8.84). Stratified analysis by tumour location showed that patients with brain tumour was more strongly associated with a higher risk of late mortality (HR 6.58; AER 6.31).

**Table 3:** Hazard ratio and absolute excess risk with 95% CI for all-cause among survivors with different types of childhood or adolescent central nervous system tumour and matched controls

Outcomes	Number of deaths		Mortality rates (per 1000 person-years)		HR	95% CI	AER	95% CI
	Survivors	Matched	Survivors	Matched				
Histology								
Medulloblastoma	61	27	15.36	1.07	17.64	10.30–30.20	14.29	8.69–23.09
Ependymoma	37	23	11.47	1.21	9.52	5.47–16.55	10.26	5.61–18.10
Meningioma	16	14	10.26	1.49	7.93	3.60–17.49	8.77	3.52–19.54
Ependymoblastoma	8	5	9.98	1.13	7.87	2.57–24.06	8.84	2.13–29.36
Astrocytoma	199	188	6.35	1.10	6.23	5.05–7.69	5.25	4.10–6.65
Craniopharyngiomas	38	32	10.23	1.56	6.20	3.82–10.07	8.67	4.84–14.81
Haemangioma	5	12	3.93	1.82	3.82	1.16–12.56	2.12	0.00–9.35
Neurinoma	19	32	5.51	1.72	3.47	1.92–6.28	3.79	1.40–8.00
Others	88	101	5.10	1.09	5.27	3.90–7.13	4.01	3.01–5.34
Location								
Brain	412	367	7.52	1.21	6.58	5.75–7.53	6.31	5.48–7.26
Spinal cord	30	33	6.87	1.36	5.80	4.01–8.40	5.50	3.36–9.02
Peripheral nerve	25	32	3.88	0.93	3.14	2.10–4.70	2.95	1.75–4.99
Others	4	2	3.98	0.38	4.48	1.67–12.01	3.60	0.66–19.66

*AER* absolute excess risk, *CNS* central nervous system, *CI* confidence interval, *HR* hazard ratio

## Discussion

In this nationwide cohort study, which to our best knowledge is the largest study on this topic, we found that long-term survivors of childhood and adolescent CNS tumour experienced a high risk of death when compared to their matched controls. The overall mortality rate declined with the increasing survival time, but it was still higher even after 30 years of follow-up. Malignant neoplasm was the leading cause of death among these long-term survivors. However, the risk of death due to malignant neoplasm underwent a sharp decline with the increasing of survival time, and it was comparable with death due to non-neoplasm cause after 30 years of follow-up. The late mortality was even more pronounced among female and survivors diagnosed at a younger age as compared to male and adolescent survivors. The risk of death was associated with specific histological types of CNS tumours with the highest risk noted among survivors with medulloblastoma.

In line with previous studies, our study suggested an elevated risk of death in long-term survivors who have ever been diagnosed with CNS tumours in childhood and adolescence even after surviving for more than 35 years when compared with the general population [3–7, 10, 12, 13]. A previous study in British Columbia indicated an excess late mortality in 5-year survivors of childhood and adolescent cancer with a standard mortality ratio of 14.0 [10]. Clinical doctors, in particular family physicians, should be aware of the high risk of late severe effects and late mortality among CNS tumour survivors who have survived for more than 5 years and were believed to reach a clinical cure. The need of increased awareness is particularly relevant during the first ten years of follow-up in the 5-year survivors. One report from Childhood Cancer Survivor Study (CCSS) suggested that only 18% of the 8522 long-term survivors with childhood cancer received risk-based and survivor-focused medical care [14]. A survey, which was conducted in the United States and Canada, found that most family physicians (85%) would be willing to care for childhood cancer survivors, but only 1% of them would be competent in caring for these patients independently [15]. Due to the lack of long-term follow-up surveillance guidelines, family physicians prefer to ensure care of these survivors in consultation with a cancer centre [15].

In the present study, the increased mortality attributed mostly to malignant neoplasm with an excess mortality rate of 3.75 per 1000 person-years, which was consistent with a few previous studies in long-term survivors of childhood cancer [3–7, 10, 12, 13]. After malignant neoplasm, long-term survivors of CNS tumour had an increased relative risk of death due to infectious and parasitic disease, diseases of the nervous system and sense organ, and diseases of the respiratory system. Of particular note was that the causes of death changed with the increasing survival time. Mortality due to neoplasm declined sharply, especially during the first 10 years of follow-up. With longer survival time, mortality caused by non-neoplastic diseases was similar as compared to malignant neoplasm (HR 1.82 for non-neoplastic diseases versus 1.76 for malignant neoplasm). The higher risk of death due to non-neoplastic diseases might be caused by tumour treatments in early life [16]. It is thus necessary to keep on monitoring for life of patients who survived over five years with an aim to minimise the excess mortality caused by late effects of tumour diagnosis and treatment.

It is worth pointing out that the risk of death was even more pronounced among female and childhood survivors as compared to male and adolescent survivors. Results from several prior studies reported a high mortality rate among female long-term cancer survivors, indicating that female survivors were at higher hazard of late mortality [3, 4, 7, 8]. Furthermore, the causes of death differed between male and female survivors, suggesting that tailored surveillance guidelines are highly needed with a consideration of gender and age at diagnosis of CNS tumour. The highest risk for death among female survivors was caused by endocrine, nutritional and metabolic diseases. Gurney et al. reported that 43% of adult survivors of childhood brain tumours had previously experienced endocrine dysfunction [17]. Evidence from survivors of childhood cancer suggested that female survivors tend to experience a higher risk of severe thyroid diseases, central precocious puberty, gonadal damage, and obesity [16, 18–20]. The risk of late mortality in childhood survivors was significantly higher than that in adolescent survivors, especially death caused by malignant neoplasm (HR 22.02 vs 8.97). Most previous studies reported the standard mortality ratio (SMR) among long-term survivors with childhood CNS tumours and the SMR ranged between 15.7 in Switzerland, 14.7 in Scotland, 12.9 in United States, and 11.5 in Great Britain [3–5, 7, 9], which suggests that cancer treatments in children had a more sustainable and serious impact on these survivors. Regarding the specific type of CNS tumour, out study found for the first time that long-term survivors with medulloblastoma had a higher risk of late mortality as compared to other types of CNS tumours.

Several strengths of this study should be mentioned. The cohort study design excluded recall bias and minimized selection bias. The accuracy of identification of cancer as well as the death of causes was guaranteed by using the Swedish registers with nationwide coverage and high sensitivity and specificity. The Swedish Cancer Registry was created in 1958 and last updated in 2016; this allowed us to follow these long-term CNS tumour survivors for several decades and to explore the patterns of death. A major limitation of this study was the lack of information on medical treatments, which made it unavailable to explore the association between medical treatments and late mortality pattern. Besides, residual confounding effect could not be totally excluded due to the lack of individual-level risk factors, such as dietary factors. However, each patient was randomly matched with cancer-free individuals’ conditional on birth year, sex, birth country, and highest education, which was supposed to partly control the potential confounding effect.

In conclusion, our study found a high mortality rate among long-term CNS tumour survivors. Although the increasing risk of death was negatively associated with the increasing survival time, the mortality rate was still higher than the general population even after 30 years of follow-up. The risk of death was even higher among female and childhood survivors, and survivors with medulloblastoma. Our data suggests that surveillance guidelines are highly needed for long-term CNS tumour survivors, and the guidelines should be tailored by gender, age at diagnosis as well as the histological type of CNS tumour to provide personalized and precision recommendations.

## Electronic supplementary material

Below is the link to the electronic supplementary material.

Electronic supplementary material 1 (TIF 177 kb)
