# Supplementary figures and images for: Mortality patterns in long-term survivors of childhood or adolescent central nervous system tumour in Sweden
Source: J Neurooncol. 2019 Nov 1;145(3):541–9. doi: 10.1007/s11060-019-03321-w (PMC6881431; doi:10.1007/s11060-019-03321-w)

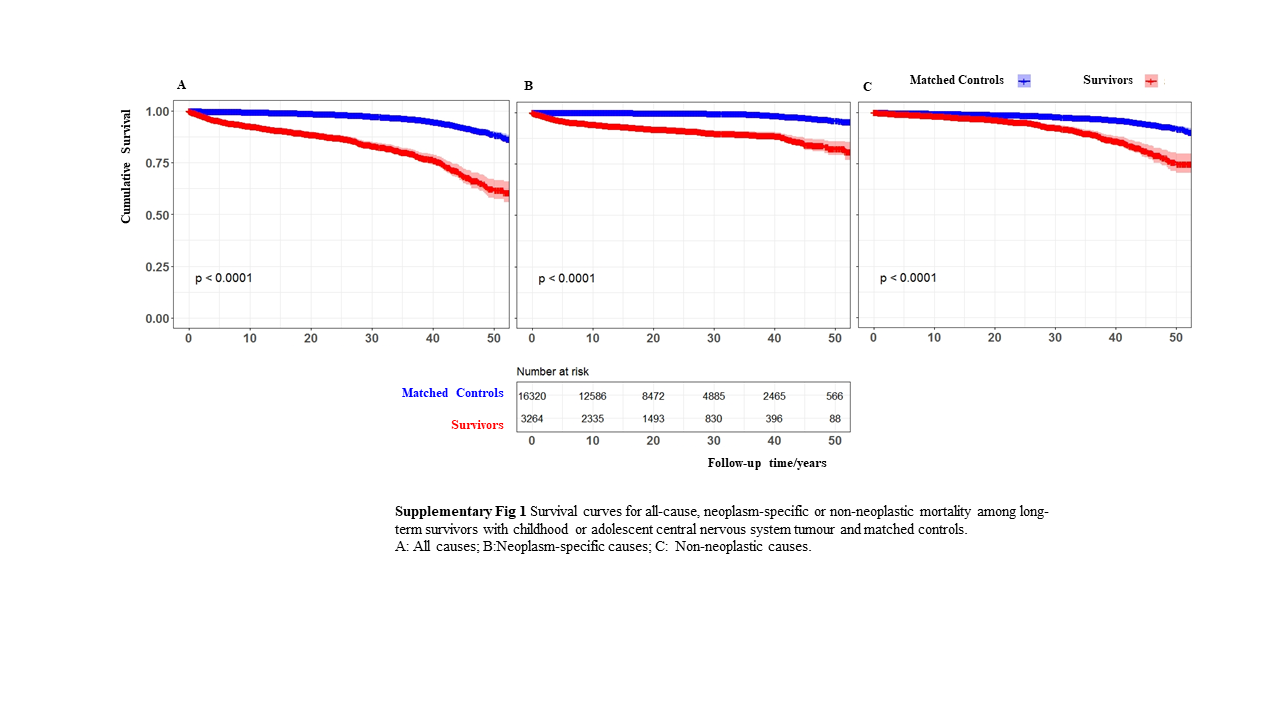

Supplement: Supplementary file 1 — Electronic supplementary material 1 (TIF 177 kb) [file 11060_2019_3321_MOESM1_ESM.tif]
